# 124. Establishment of a Post-Influenza Aspergillosis Model in Corticosteroid-Immunosuppressed Mice

**DOI:** 10.1093/ofid/ofab466.124

**Published:** 2021-12-04

**Authors:** Sebastian Wurster, Jezreel Pantaleón García, Nathaniel D Albert, Scott Evans, Dimitrios P Kontoyiannis

**Affiliations:** The University of Texas MD Anderson Cancer Center, Houston, TX

## Abstract

**Background:**

Post-influenza aspergillosis (PIA) is a feared complication in patients with severe influenza, especially those receiving corticosteroids. However, validated murine models of PIA in a background of corticosteroid immunosuppression are lacking, compounding efforts to better characterize the immunopathology and treatment of this emerging entity.

**Methods:**

8-week-old female BALB/c mice were infected with ~5% of the lethal dose of a mouse-adapted influenza A/Hong Kong/1968 (H3N2) strain (flu), delivered by aerosolization, versus control (aerosolized saline). Mice then received two intraperitoneal injections of 10 mg cortisone acetate (CA) or mock injections on days 5 and 8 after flu infection. On day 9, mice were intranasally challenged with 50,000 *A. fumigatus* AF-293 conidia or mock-infected with saline. Survival was monitored until day 16 and infection severity was scored using the modified murine sepsis score (MSS, 0 = healthy to 3 = moribund). Pulmonary fungal burden was determined by an 18S quantitative PCR assay on day 16 or upon death. 15-16 mice per group were assessed across 3 independent experiments.

**Results:**

Flu infection alone caused modest early morbidity, followed by full recovery of the mice until day 16. Treatment with CA after flu infection led to 12% mortality and increased morbidity that persisted until day 16 (median MSS = 0.8). Similarly, mice infected with AF after CA treatment had 12% mortality and a median MSS of 0.7. Combination of all 3 challenges (flu, CA, and AF) led to 40% mortality and severe morbidity in surviving mice (median MSS = 2.7). Likewise, prior flu infection of CA-treated, AF-infected mice increased the pulmonary fungal burden from 27k to 80k median conidial equivalents. In contrast, mice not receiving CA treatment showed consistently low morbidity (median day-16 MSS = 0.5) and minimal fungal burden after AF challenge, regardless of prior flu infection.

**Conclusion:**

We have established a model of PIA in CA-immunosuppressed mice that underscores the detrimental effect of corticosteroid therapy on the outcomes of PIA. In the future, we will employ this model to study the impact of various pharmacological interventions on the natural history of PIA in the background of corticosteroid immunosuppression.

**Disclosures:**

**Dimitrios P. Kontoyiannis, MD**, **Astellas** (Consultant)**Cidara Therapeutics** (Advisor or Review Panel member)**Gilead Sciences** (Consultant, Grant/Research Support, Other Financial or Material Support, Honoraria)

